# Improving pediatric procedural skills and EPA assessments through an acute care procedural skills curriculum

**DOI:** 10.1371/journal.pone.0306721

**Published:** 2024-08-30

**Authors:** Maaz Mirza, Elif Bilgic, Ronish Gupta, Quang N. Ngo, Karen Forward

**Affiliations:** 1 Department of Pediatrics, McMaster University, Hamilton, Ontario, Canada; 2 Division of Pediatric Emergency Medicine, McMaster University, Hamilton, Ontario, Canada; 3 McMaster Education Research, Innovation and Theory (MERIT) Program, McMaster University, Hamilton, Ontario, Canada; 4 Division of Pediatric Critical Care Medicine, McMaster University, Hamilton, Ontario, Canada; NYU Grossman School of Medicine: New York University School of Medicine, UNITED STATES OF AMERICA

## Abstract

**Introduction:**

Acute procedural skill competence is expected by the end of pediatric residency training; however, the extent to which residents are actually competent is not clear. Therefore, a cross-sectional observational study was performed to examine the competency of pediatric residents in acute care procedures in emergency medicine.

**Materials and methods:**

Pediatric residents underwent didactic/hands-on “Acute Procedure Day” where they performed procedures with direct supervision and received entrustable professional activity (EPA) assessments (scores from 1–5) for each attempt. Procedures included: bag-valve mask (BVM) ventilation, intubation, intraosseous (IO) line insertion, chest tube insertion, and cardiopulmonary resuscitation (CPR) with defibrillation. Demographic information, perceived comfort level, and EPA data were collected. Descriptive statistics and Pearson correlation for postgraduate year (PGY) versus EPA scores were performed.

**Results:**

Thirty-six residents participated (24 PGY 1–2, and 12 PGY 3–4). Self-reported prior clinical exposure was lowest for chest tube placement (n = 3, 8.3%), followed by IOs (n = 19, 52.8%). During the sessions, residents showed the highest levels of first attempt proficiency with IO placement (EPA 4–5 in 28 residents/33 who participated) and BVM (EPA 4–5 in 27/33), and the lowest for chest tube placement (EPA 4–5 in 0/35), defibrillation (EPA 4–5 in 5/31 residents) and intubation (EPA 4–5 in 17/31). There was a strong correlation between PGY level and EPA score for intubation, but not for other skills.

**Discussion:**

Entrustability in acute care skills is not achieved with current pediatrics training. Research is needed to explore learning curves for skill acquisition and their relative importance.

## 1. Introduction

The literature regarding procedural skills training and assessment for pediatric residents is limited [1]. There are currently no evidence-supported guidelines suggesting the best path towards procedural skills competency for residents [2]. This poses a significant barrier in the development, delivery, and improvement of procedural training curricula. Physician trainees are less likely to receive formal training and/or supervision for completing procedural skills than their nursing counterparts and report feeling inadequately prepared [1, 3]. Thus, the creation of opportunities for residents to gain competency in procedural skills should be addressed within pediatric residency training.

The Royal College of Physicians and Surgeons of Canada (RCPSC) has developed key Entrustable Professional Activities (EPAs) focused on acute care procedures and skills in which pediatric residents must show competence by the end of training [4]. These skills include: Intraosseous placement, chest tube placement, cardiopulmonary resuscitation, and airway management [5]. According to a 2018 survey of 481 Canadian general pediatricians, these procedures were felt to be important; however, they were performed “yearly” or “rarely” with limited opportunities to maintain procedural preparedness [6]. Among surveyed Canadian pediatric residency program directors, there was a significant difference between perceived importance of common procedures and resident preparedness [2].

Simulation-based medical education lends itself well to skill acquisition and assessment [7]. The RCPSC has outlined specific assessment plans for each EPA, whereby residents are required to receive a specific number of EPA assessments. Though currently, programs primarily focus on completion of EPA assessments for encounters in the clinical setting, in-person simulations have been brought up as an additional setting to conduct EPA assessments [8]. According to the RCPSC, it is specifically written that some EPA assessments can be completed in the simulation setting, which is especially important for procedures that are rarely encountered clinically [5].

Therefore, the purpose of this study was to present a cross-sectional observation of the entrustability of pediatric residents for 6 procedural skills in emergency medicine in the simulation setting.

## 2. Materials and methods

Programmatic assessment component of the Core Components Framework for Evaluating Implementation of CBD Programs, which focuses on assessment practices that support and document the developmental acquisition of competencies, is the guiding framework for our research [9]. A mixed didactic and hands-on acute care procedural skills curriculum was developed for core acute care procedure skills in Pediatrics, including chest tube placement, Intraosseous (IO) needle insertion, cardiopulmonary resuscitation (CPR), defibrillation, bag-valve mask ventilation (BVM), and intubation. These skills had been identified as important for procedural competence as per the “*Pediatrics Competencies* 2021” by the RCPSC and based on the findings in the literature [5]. The curriculum was implemented in Fall 2022.

### 2.1 Eligibility criteria

Participants included all pediatric residents at McMaster University in their 1st through to 4th year of postgraduate-year (PGY) training.

### 2.2 Instructional methods

For each procedure, instructional methods were interactive and included 1-hour educational sessions covering indications, contraindications, complications, and procedural steps. Residents were provided with the milestones to procedural success for their review. Residents subsequently practiced each procedural skill under the direct supervision of a pediatric acute care staff physician or subspecialty trainee during one of two identical "Acute Care Procedure Day(s)” occurring 1–2 weeks apart. Education sessions and procedure days were held between 5 October to 2 November 2022. An EPA assessment was completed for each attempt (score of 1–5). Due to time constraints, not all residents were able to receive an EPA assessment, but all were given the opportunity to practice the skills.

### 2.3 Assessment

Residents completed a Google-form-based survey pre and post the procedure skills day. The pre-session survey collected information such as PGY level, previous experience with the six procedures (Yes/No), number of previous procedures performed both clinically and in simulation, and self-reported comfort with carrying out each procedure on a 10-point Likert scale (See S1 File). The post-session survey collected the number of attempts at a procedure during the simulation session, and post-session comfort.

### 2.4 Data analysis

Descriptive statistics and Pearson correlation (above 0.7 is considered as high positive correlation) for PGY level versus EPA scores were performed.

### 2.5 Consent and ethics

The data was originally collected under a Quality Improvement Initiative of the Department of Pediatrics, with confirmation that REB review was not required for this work provided by the Hamilton Integrated Research Ethics Board (HiREB). Nonetheless, we informed the residents of the initiative, and none of the residents were minors. HiREB has approved the re-use of this anonymized data for research purposes and dissemination in this manuscript.

## 3. Results

Thirty-six residents participated; 24 (67%) in PGY 1–2, and 12 in PGY 3–4 (33%). All 36 residents completed the pre-skills day questionnaire and of those, 34 (94%) also completed the post-skills day questionnaire; the two residents who did not complete the post-skills day questionnaire had to leave early. Reported prior clinical experience was lowest for placement of chest tubes (n = 3, 8.3%), followed by IOs (n = 19, 52.8%). The self-perceived comfort level (Table 1) with each procedure prior to participation, as measured by a 10-point Likert scale, was highest for BVM (7.11), and lowest for chest tube placement (2.31). The magnitude of change in self-reported comfort after participating in the skills day was highest for procedures with the lowest initial comfort levels, with chest tube (+2.72) and IO insertion (+2.47) having the highest.

**Table 1:** Average self-reported comfort level and first attempt entrustment levels.

	*Chest Tube*			*IO Insertion*			*CPR*			*BVM*			
*PGY Level*	*Pre*	*Post*	*Δ*	*Pre*	*Post*	*Δ*	*Pre*	*Post*	*Δ*	*Pre*	*Post*	*Δ*	
**Self-Reported Comfort** *													
1	2.00	5.92	3.92	4.92	7.08	2.16	6.67	7.25	0.58	6.83	8.00	1.17	
2	1.10	3.64	2.54	4.40	8.00	3.60	6.90	8.55	1.65	6.50	8.73	2.23	
3	3.38	5.00	1.62	5.25	7.14	1.89	7.13	8.43	1.30	7.38	9.00	1.62	
4	3.80	6.25	2.45	5.80	7.50	1.70	6.40	7.25	0.85	8.60	9.00	0.40	
*Overall*	2.31	5.03	2.72	4.97	7.44	2.47	6.80	7.91	1.11	7.11	8.00	0.89	
***First Attempt Entrustment*** **		*Chest Tube*			*IO Insertion*			*CPR*			*BVM*		*Intubation*
1		2.85			5.00			3.67		3.85			3.00
2		2.36			4.00			3.56		3.91			3.36
3		2.33			4.75			4.33		4.43			4.29
4		2.80			4.80			3.75		4.60			4.60
*Overall*		2.60			4.61			3.72		4.08			3.68
*Correlation with PGY Level*		-0.16			-0.10			0.12		0.44			0.77

* Resident comfort with procedures across PGY levels before and after the procedural skills day

**Resident EPA scores for first attempt across PGY levels

First attempt entrustment (Table 1), defined as an EPA score of 4 or 5 on the first attempt, varied greatly both between and within PGY levels. Residents showed the highest levels of first attempt entrustment with IO placement (EPA 4 or 5 in 28 residents out of 33 who received an EPA assessment), BVM (27/33), and CPR (16/25), and lowest for chest tube placement (0/35), defibrillation (5/31 residents), and intubation (17/31).

Correlation for EPA scores on the first attempt of a procedure with level of training showed a strong correlation between PGY level and EPA score for intubation, and no correlation for the other procedures (Table 1).

## 4. Discussion

In this study, after participation in an acute care skills curriculum with hands-on simulation experience, the majority of residents showed entrustability for IO placement and BVM skills; however, single-session entrustment was low for both CPR with defibrillation and intubation, with no residents being entrustable for placement of chest tubes. Entrustability did not appear to correlate with year in training with the exception of intubation.

We would expect entrustability to be high for skills that are both easy to learn and/or exposure is high. Unsurprisingly, BVM skills are amongst the highest entrustability of the procedures we assessed as pediatricians attend births often and the proportion of babies requiring minimal resuscitation up to BVM is frequent enough that it is reasonable to conclude clinical exposure to this skill alone is enough to maintain competence [10, 11]. IO placement has been considered the standard for pediatric patients for whom IV access is difficult and has been made easier by the advent of semi-automatic powered devices [12, 13]. In fact, once taught, it appears that decay is not as rapid as in other skills, which may explain why the entrustability of this skill remains relatively high amongst this cohort of pediatrics residents [14]. With respect to CPR, while not a difficult skill to learn, it is seldom performed in pediatrics and the skill decay rate is well described beyond 6 months without refreshing [15].

Lack of correlation between procedural skills entrustability and PGY level is reflective of the relative rarity these skills are encountered in, with the exception of intubation. These results push us to be more deliberate in how we set up our training programs and prepare our residents for clinical practice. It also forces us to reassess whether certain skills that are difficult to achieve clinically are relevant. A needs assessment done by White et al. showed wide variability in skills recommended by the RCPSC among practicing pediatricians and a negative correlation between procedures being performed and difficulty maintaining proficiency in a skill [6]. Our results would support these findings.

There are several challenges and limitations associated with implementing a procedure-based simulation curriculum:

1. Human resources: Procedural teaching curricula require a team of qualified and experienced instructors with expertise in simulation-based training, curriculum design, and the specific clinical procedures being taught. However, finding and retaining qualified instructors can be difficult.
2. Financial & Physical resources: Simulation-based training requires significant financial investments (i.e. equipment, supplies, administrative support, instructors, and facilities). These costs can be prohibitive for smaller healthcare organizations or those with limited budgets.
3. Time constraints: Significant time commitments are required for both instructors and residents for curriculum design and development as well as resident learning and feedback which takes place away from the workplace.

Finally with respect to feasibility, it is important to prioritize procedures that are both necessary and rare to inform targeted training. One way to address the feasibility challenges that come with in-person simulations is to explore the role of virtual simulations, which are flexible and less resource intensive [16, 17]. Nonetheless, virtual and in-person simulations could be used as an adjunct to the clinical setting to increase exposure and help residents achieve entrustment on the EPAs.

We recognize that the generalizability of these findings is limited as this represents a single center in Canada. However, given the standardization of training programs by the RCPSC, we feel that the concern about variability in attainment of procedural competence is likely shared amongst other programs [18].

## 5. Conclusion

Presently, pediatric residents are not achieving entrustability in acute procedural skills. The findings likely reflect rare opportunities to learn these procedures in the clinical environment. Simulation education continues to be a useful tool in developing familiarity with infrequently encountered clinical scenarios and procedures. More studies are, however, required to determine the appropriate training schedule and methods for achieving procedural competency, which may in fact differ by individual procedure. Furthermore, given the resource intensiveness of simulation education, identifying procedures with the highest potential clinical yield (e.g., high acuity, and low but not zero frequency) will help maximize the return on simulation investment.

## Supporting information

S1 File Pre- and post-session surveys. (PDF)
