# Supplementary material for: Improving pediatric procedural skills and EPA assessments through an acute care procedural skills curriculum
Source: PLoS One. 2024 Aug 30;19(8):e0306721. doi: 10.1371/journal.pone.0306721 (PMC11364283; doi:10.1371/journal.pone.0306721)
Supplement: S1 File — (PDF) [file pone.0306721.s001.pdf]

Supplemental 1 File. Pre- and Post-Session Surveys

Pre-Session Survey

Demographics

Name: __________________

Level of training

PGY1

PGY2

PGY3

PGY4

Date: DD/MM/YY

Chest Tube Insertion

Previous experience with chest-tube placement

- Yes
- No

Approximate number of chest tubes placed clinically?

- 0
- 1-2
- 3-4
- 5-6
- 7-8
- 9+

Approximate number of chest tubes placed in simulation?

- 0
- 1-2
- 3-4
- 5-6
- 7-8
- 9+

What is your comfort with performing a CHEST TUBE clinically on a scale of 1-10

Not at all comfortable - 1 2 3 4 5 6 7 8 9 10 - Completely comfortable & confident

Intraosseous (IO) Needle Insertion

Previous experience with intraosseous (IO) needle insertion

- Yes
- No

Approximate number of IO needles placed clinically?

- 0
- 1-2
- 3-4
- 5-6
- 7-8
- 9+

Approximate number of IO needles placed in simulation?

- 0
- 1-2

- 3-4
- 5-6
- 7-8
- 9+

What is your comfort with performing IO NEEDLE PLACEMENT clinically on a scale of 1-10

Not at all comfortable - 1 2 3 4 5 6 7 8 9 10 - Completely comfortable & confident

Cardiopulmonary Resuscitation (CPR)

Previous experience with cardiopulmonary resuscitation (CPR)

- Yes
- No

Approximate number of times you performed CPR clinically?

- 0
- 1-2
- 3-4
- 5-6
- 7-8
- 9+

Approximate number of times you performed CPR in simulation?

- 0
- 1-2
- 3-4
- 5-6

- 7-8
- 9+

What is your comfort with performing CPR clinically on a scale of 1-10

Not at all comfortable - 1 2 3 4 5 6 7 8 9 10 - Completely comfortable & confident

Bag-Valve Mask Ventilation (BVM)

Previous experience with bag-valve mask ventilation (BVM)

- Yes
- No

Approximate number of times you performed BVM clinically?

- 0
- 1-2
- 3-4
- 5-6
- 7-8
- 9+

Approximate number of times you performed BVM in simulation?

- 0
- 1-2
- 3-4
- 5-6
- 7-8
- 9+

What is your comfort with performing BVM clinically on a scale of 1-10

Not at all comfortable - 1 2 3 4 5 6 7 8 9 10 - Completely comfortable & confident

Post-Session Survey

Demographics

Name: _________________

Level of training

PGY1

PGY2

PGY3

PGY4

Date: DD/MM/YY

Chest Tube Insertion

Approximate number of chest tubes placed in simulation?

- 0
- 1
- 2
- 3
- 4
- 5+

What is your comfort with performing a CHEST TUBE clinically on a scale of 1-10

Not at all comfortable - 1 2 3 4 5 6 7 8 9 10 - Completely comfortable & confident

Intraosseous (IO) Needle Insertion

Approximate number of IO needles placed in simulation?

- 0
- 1
- 2
- 3
- 4
- 5+

What is your comfort with performing IO NEEDLE PLACEMENT clinically on a scale of 1-10

Not at all comfortable - 1 2 3 4 5 6 7 8 9 10 - Completely comfortable & confident

Cardiopulmonary Resuscitation (CPR)

Approximate number of times you performed CPR in simulation?

- 0
- 1
- 2
- 3
- 4
- 5+

What is your comfort with performing CPR clinically on a scale of 1-10

Not at all comfortable - 1 2 3 4 5 6 7 8 9 10 - Completely comfortable & confident

Bag-Valve Mask Ventilation (BVM)

Approximate number of times you performed BVM in simulation?

- 0
- 1
- 2
- 3
- 4
- 5+

What is your comfort with performing BVM clinically on a scale of 1-10

Not at all comfortable - 1 2 3 4 5 6 7 8 9 10 - Completely comfortable & confident
